# Co-Opting Host Receptors for Targeted Delivery of Bioconjugates—From Drugs to Bugs

**DOI:** 10.3390/molecules26051479

**Published:** 2021-03-09

**Authors:** Kristen M. Tummillo, Karsten R.O. Hazlett

**Affiliations:** 1 Department of Regenerative and Cancer Cell Biology, Albany Medical College, Albany, NY 12208, USA; kristen.tummillo@admerahealth.com; 2 Admera Health, South Plainfield, NJ 07080, USA

**Keywords:** antibody–drug conjugates, drug delivery, vaccines, antibiotics, conjugated vaccines, targeted vaccines, bioconjugation, nanoparticles

## Abstract

Bioconjugation has allowed scientists to combine multiple functional elements into one biological or biochemical unit. This assembly can result in the production of constructs that are targeted to a specific site or cell type in order to enhance the response to, or activity of, the conjugated moiety. In the case of cancer treatments, selectively targeting chemotherapies to the cells of interest limit harmful side effects and enhance efficacy. Targeting through conjugation is also advantageous in delivering treatments to difficult-to-reach tissues, such as the brain or infections deep in the lung. Bacterial infections can be more selectively treated by conjugating antibiotics to microbe-specific entities; helping to avoid antibiotic resistance across commensal bacterial species. In the case of vaccine development, conjugation is used to enhance efficacy without compromising safety. In this work, we will review the previously mentioned areas in which bioconjugation has created new possibilities and advanced treatments.

## 1. Introduction

The process of conjugation has allowed scientists to improve therapies and vaccinations by linking therapeutic agents or protective antigens to targeting moieties that focus delivery of the cargo. Increasing the specificity of therapies promotes concentration of the agent which consequently reduces the needed systemic dose and minimizes side effects. Targeting of prophylactics and therapies can be achieved by fusing distinct functional moieties; a process often termed bioconjugation. Common methods of bioconjugation include genetic fusion, chemical conjugation, and in vivo bioconjugation; examples of each will be discussed in this review. Bioconjugation is broadly utilized from basic pre-clinical development to current clinical use and impacts medical disciplines from cancer, to neuropathies, to infectious disease.

In the case of treating cancer patients, systemic administration of chemotherapy regularly causes deleterious side effects and has limited efficacy; however, great strides have been made in limiting side effects and enhancing efficacy by specifically targeting therapies. Through targeted drug delivery, drugs can be concentrated in the desired tissue and specific anti-tumor cells can be activated [1,2,3,4,5]. The targeting concept has also been applied to neurodegenerative diseases with the goal of suppressing pathological immune responses and/or promoting repair pathways [6,7]. The ability to target antibiotics is a promising development as drug resistant pathogens have become increasingly prevalent and indiscriminate application of antibiotics promotes the development and spread of antibiotic resistance [8,9]. Further, conjugated vaccines have also been developed for many pathogenic microbes; the importance of the latter is glaringly highlighted by the COVID-19 pandemic.

The goal of this mini-review is to succulently highlight the breadth of receptor-targeting approaches employed by bioconjugate therapies and bioconjugate vaccines—both nascent and mature. To this end, we are skimming the surfaces of many broad fields (cancer, neuropathology, infectious disease) to show-case the diversity of creative targeting modalities.

## 2. Targeted Therapies

### 2.1. Cancer Therapies

Chemotherapy agents inhibit cell growth and division by inducing DNA damage, preventing DNA replication, and disrupting mitosis. Inhibiting the process of cell division is aimed at destruction of rapidly growing cancer cells, however, the division of rapidly dividing healthy cells is also susceptible to growth inhibition and killing by chemotherapy treatments. This leads to deleterious systemic side effects, such as hair loss and significant disruption of the gastrointestinal tract and immune system [10,11]. The severe side effects and poor therapeutic window(s) of conventional chemotherapy can leave patients weighing the risks and benefits of such treatments versus living with cancer. Therefore, there has been a great push for development of increased specificity and efficacy in cancer treatments. Conjugation of chemotherapy drugs to antibodies (Ab) can allow for direction of a cytotoxin to cancer cells; reducing side effects in healthy tissues (Table 1). Drugs can be linked to monoclonal antibodies (mAb) via cleavable peptides which release the active drug once it is taken up by cells into acidified compartments [12,13]. A limiting factor in developing these therapies can be discovery of cancer-specific antigens (Ag) to which the Ab-conjugated therapy may be targeted.

LIV-1 is an estrogen-regulated zinc transporter that is highly expressed in most breast cancers while its expression levels in other tissues is low [14,15]. Therefore, targeting chemotherapies to breast cancer cells has been achieved by conjugating the drug to humanized anti-LIV-1 Abs [15,16,17]. Another Ag that has been targeted in breast cancer is HER2 (human epidermal growth factor receptor-2), which when targeted, may concentrate therapies even in HER2-low cancers [15,18]. Many other Ab-drug conjugates are in the clinical stages of development including those specific for CD33 which is a sialic acid receptor that is over expressed in acute myeloid leukemia [19,20] and those specific for STEAP1 (six transmembrane epithelial antigen of the prostate 1) which is over expressed in prostate cancers [21]. Overall, the conjugation of cytotoxins to cancer-specific Abs has increased drug delivery to primary tumors and metastatic sites as well as cancers of the blood. Ab-drug conjugates have been approved by the FDA for treatment of Hodgkin lymphoma [22], HER2-positive breast cancer [23], acute myeloid leukaemia [24], and lymphoblastic leukaemia [25]; many others are currently in clinical trials for various diseases [26].

In addition to targeting receptors for location-specific concentration of therapies, controlled release of therapeutic substances also improves their efficacy and reduces off target effects. In some cases, non-internalizing targets on tumor cells are used to deliver Ab-drug conjugates to the tumor and any unbound complexes are cleared; next an activator is administered to release the drug from the antibody, allowing it to defuse into local tumor cells. This method reduces toxic side effects on healthy cells and increases efficacy of the drugs. It also allows for use of tumor targets that are not known to enhance uptake when engaged. This extracellular release of active drug can involve bioorthogonal, or “click-to-release”, reactions where a reaction between tetrazine and *trans*-cyclooctenes cause cleavage of allylic carbamates and release of the conjugated cytotoxin [27,28,29]. Work from the Robillard lab has demonstrated release of an antimitotic toxin from a diabody specific for tumor-associated glycoprotein-72 (gp72) when the tetrazine activator was administered to mice. Treatment with this technique resulted in increased anti-tumor activity in both ovarian and colon carcinoma models in mice; this was achieved without signs of off-target toxicity as the drug was selectively concentrated at the tumor site and was absent or present at low levels in other tissues [30]. Another form of “click to release” involves an isonitrile-based cleavage that was discovered by the Franzini lab and involves the rapid bioorthogonal removal of a 3-isocyanopropyl masking group through reaction with tetrazines [31,32,33]. Collectively, this method of selected drug release reduces side effects on healthy cells and increases potency of anti-cancer therapies.

### 2.2. Central Nervous System Therapies

Alzheimer’s disease is the most common form of dementia in the United States, where there are currently over 5 million people living with the disease [53]. Brain pathology characteristically involves the development of amyloid beta plaques, aggregated tau protein, neurofibrillary tangles, and a variety of neurological defects. As researchers have discovered more about Alzheimer’s disease, they have also unveiled promising targets for therapies [6]. However, a major limitation on delivery of neurodegenerative therapies is bioavailability and crossing the blood-brain-barrier (BBB). Fan et al. developed a brain-targeted nanoparticle (NP) that increases the delivery of curcumin, an anti-inflammatory compound [36]. Targeting of the curcumin-loaded NPs to the brain was achieved through conjugation of the particles to B6 peptides. B6 is a short peptide that binds the transferrin receptor (TfR) which is expressed on endothelial cells of the BBB and facilitates transcytosis; targeting to this receptor increases BBB-permeability and availability of B6-conjugates in the brain [36,54,55,56]. Administration of the B6-conjugated curcumin-loaded NP to mice improved their memory and spatial learning which has promise to extend to treatment of humans with neurodegenerative diseases [36].

Parkinson’s disease is another common neurodegenerative disorder that is characterized by the slowing of movements and the presence of a resting tremor [7,57]. As with Alzheimer’s disease, delivery of therapeutics for Parkinson’s disease also requires crossing the BBB. Rusiecka et al. enhanced the delivery of dopamine to the brain through conjugation to the cell-penetrating peptide TP10 [37] which is derived from wasp venom [58]. This heightened cell permeability improves delivery of TP10-conjugates to the brain and in the case of dopamine, delivers it in a form that has a high affinity for its receptor [37]. It should be noted that the lipophilic peptides such as TP10 are not inherently BBB specific and additional BBB-specific targeting modalities might enhance the specificity of this approach.

Additional examples of targeted CNS therapies include those focused on demyelination (a hallmark of multiple sclerosis-MS) and post-stroke, neuro-regeneration [38,39]. The aptamer LJM-3064 is a small (40 nt, ~13 kDa) single stranded DNA molecule selected in a screen for myelein-binding activity [59]. Tetramers of LJM-3064 administered to mice in a viral encephalitis model displayed significant remyelination of the spinal cord. The results indicate that the myelin-binding aptamer, when multimerized, has inherent pro-myelinogenic properties. LJM-3064 has subsequently been used to decorate the surfaces of anti-inflammatory exosomes (derived from mesenchymal stem cells). The aptamer-exosome bioconjugate reduced both CNS inflammation and demyelination in a murine experimental autoimmune encephalomyelitis (EAE) model of MS [38].

Neuro-regeneration by adult neural stem cells holds tremendous medical potential but has been limited, particularly in the context of ischemic stroke, by poor survival of migrating neuroblasts. In attempt to activate pro-survival pathways in these cells, the Akt-activating drug SC-79 has been encapsulated in NP decorated with Ab specific for a neural cell adhesion molecule (PSA-NECM). When the drug-loaded conjugates (α-PSA-NECM-NP-SC-79) were instilled into murine lateral ventricles, the authors noted activation of Akt in neuroblasts within in the subventricular zone [39]. Collectively, the ability to target conjugates for enhanced passage across the BBB and/or to specific sites of pathology shows promise for enhancing treatment of brain-specific diseases.

### 2.3. Antimicrobials

Since the discovery of penicillin by Alexander Fleming in the early 1900’s, antibiotics have been used to treat countless infections and have greatly improved medical care. However, emerging bacterial resistance to antibiotics is widely recognized and has hindered the effective use of many compounds [60,61]. The emergence of new and more extensively drug-resistant bacteria have encouraged the discovery of novel antibiotic compounds as well as targeted delivery of existing antibiotics which promotes more direct and concentrated administration of a drug [62]. By focusing delivery of the drug to the pathogen which is causing disease, the exposure of commensal microbes to antibiotic agents is reduced. This is important to prevent development of antibiotic resistance; in many cases the antibiotic activity of a drug is broad and sub-lethal exposure permits bacteria to enhance their drug resistance mechanisms. Therefore, targeting an antibiotic to a specific site or pathogen can both reduce generation of drug resistant microbes while leaving endogenous commensal (“good”) bacterial populations intact.

One method of targeting delivery of antibiotics and limiting off-target effects is to conjugate the antibiotic to a pathogen-specific Ab. Lehar et al. conjugated the antibiotic rifalogue to an Ab specific for MRSA (methicillin-resistant *Staphylococcus aureus*) and showed increased killing of MRSA in a murine bacteremia model. In this case, the antibiotic was not active until the conjugate-opsonized bacteria were engulfed in a phagosome, it was then activated by intracellular proteases and able to kill the bacteria within the host cell [40]. This method not only concentrates the antibiotic at the location of the pathogen, but also reduces exposure of commensal bacteria to the drug which would have the potential to induce antibiotic resistance mechanisms.

Similarly, targeted NPs and virus-like particles (VLPs) have also been used to concentrate antibiotic delivery. NPs enhance bioavailability, solubility, stability, and controlled release of different substances. In addition, due to their size, NPs are taken up by phagocytic mechanisms versus the passive entry of smaller molecules such as free drug [63,64]. Antibiotic-decorated particles have been shown to increase the amount of antibiotic internalized by lung-resident macrophages; therefore, concentrating the drug and potentially increasing effectiveness of treatments against stubborn pulmonary bacterial infections [65]. Gold NPs have also been utilized to enhance efficacy of antibiotics and reduce the necessary dose [66,67,68]. Work by Pornpattananangkul et al. utilized antibiotic-loaded gold NP that would release the drug upon contact with the pore-forming α-toxin of MRSA [41]. Since the particle did not liberate the antibiotic without the presence of toxin, the drug would only be released in sites near to the infection. Again, this concentrates the drug, increasing its activity and reducing off-target effects.

## 3. Targeted Vaccinations

**Figure 1:**
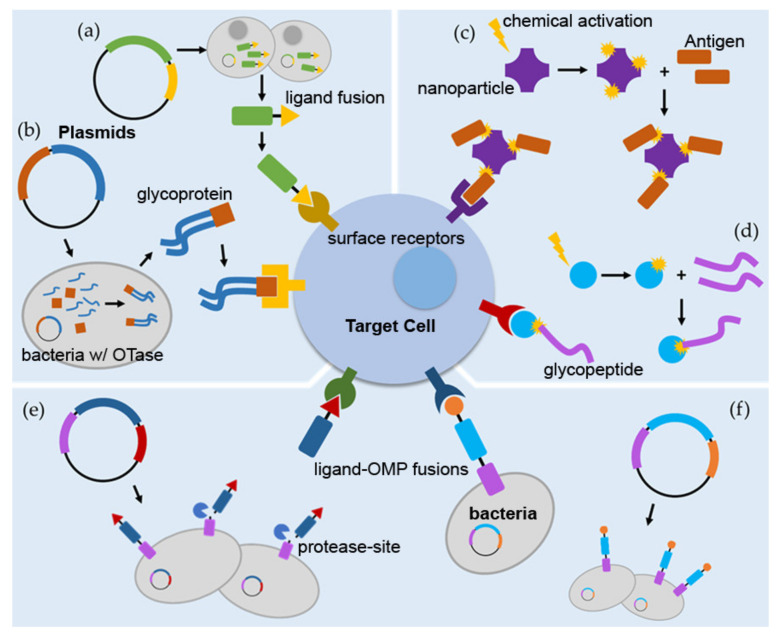
Schematic of targeted conjugate vaccines. (**a**) A plasmid encoding a protein subunit conjugate is transfected into mammalian cells which secrete the fusion protein for subsequent purification and administration as a vaccine. Modeled after HA targeting to Xcr1 on DCs [44]. (**b**) A plasmid encoding a peptide carrier and capsular PS synthases is transformed into an oligosachride transferase (OTase)—engineered strain of *E. coli* which assembles the glycoconjugate which is subsequently purified for use as a vaccine. Modeled after production of a *Kp* capsular PS-*Pa* exotoxin A glycoconjugate vaccine [46]. (**c**) NPs are chemically activated to facilitate conjugation of Ag; loaded NPs are then administered as the vaccine. Modeled after activated amines to conjugate Pfs25 and generate a malaria vaccine [47]. (**d**) Glycoconjugate vaccines produced by chemically activating a peptide carrier, linking the purified components, and administering the conjugate as a vaccine. Modeled after generation of the meningitis vaccine [45]. (**e**) A plasmid encoding a targeted protein subunit conjugate that is anchored to an OMP is transformed into bacteria which are administered as a live vaccination. The conjugate protein is trafficked to the OM and cleaved from the OMP anchor. Modeled after CR-targeted *Ts* Ag, produced by the engineered *Salmonella* vaccine strain [52]. (**f**) A plasmid encoding an OMP-anchored conjugate (lacking a cleavage site) is expressed in bacteria, effectively targeting the whole bacterial cell to immune cells when administered as a vaccine. Modeled after targeting *Ft* to CRs [51].

Vaccinations have been used for centuries in order to protect populations against disease-causing microorganisms. In fact, the process of intentionally exposing an individual to a less virulent form of a pathogen has led to eradication of the disfiguring and deadly disease, small pox [69]. However, while this vaccine prevented later contraction of small pox, it initially caused mild disease as it involved administration of virulent cow pox virus [70]. Vaccine development has since evolved with an interest in increasing the safety of vaccinations without sacrificing protective efficacy. Inactivation of pathogens such as influenza [71] and cholera [72] greatly increase their safety for use as a vaccines. Safety is even further improved in vaccines against Hepatitis B [73], diptheria, and tetanus [74]; utilizing purified proteins or inactivated toxins in subunit vaccines: HBsAg and DTaP, respectively. Increasing the safety of these vaccines comes at a trade off with reduced efficacy since there is no live infection generating danger signals. In some cases, immune stimulation is restored through the addition of an adjuvant such as alum [75,76]. However, many adjuvants cause off-target effects, including unwanted activation of bystander cells and potential induction of autoimmunity [77,78]. These drawbacks to adjuvants have encouraged the development of adjuvant-independent enhancement of vaccines; this has been achieved through fusion of Ag to stimulatory or targeting molecules. Research aimed at generating a vaccine against a potential biological weapon, *Francisella tularensis* (*Ft*), has shown that opsonizing inactivated *Ft* with α-*Ft* Abs targets the bacteria to Fc receptors on immune cells. This interaction stimulates uptake and presentation of Ag, activation of immune cells, and ultimately results in production of an enhanced protective immune response against virulent *Ft* challenge [79,80,81]. Similar methods of targeting to host cells have been achieved through bioconjugation; we have diagrammed several of these in Figure 1.

### 3.1. Protein Subunit Vaccine Conjugates

Directing protein Ag to Ag-presenting cells (APCs) such as dendritic cells (DCs), increases both cellular and humoral immune responses. DCs phagocytose Ag and subsequently process and present peptides via both major histocompatibility complex-I (MHCI) and -II (MHCII), allowing for involvement of a variety of downstream cells. One example of targeting Ag to DCs is accomplished through the activating receptor, Clec9A (C-type lectin domain family 9 member A) [48,82,83]. Park et al. accomplished fusion of the influenza Ag M2e to α-Clec9A Ab through genetic engineering of a plasmid. Plasmids encoded the heavy and light chains of α-Clec9A Ab, a flexible glycine linker, and three repeats of the M2e peptide. The plasmids were then transfected into Freestyle 293F cells for expression of the Ag-Ab conjugates. The secreted Ag-Ab fusions were purified via protein G affinity chromatography. Ultimately, administration of the targeted Ags to mice resulted in increased specific immunoglobulin responses and increased protection against lethal influenza infection compared to Ag conjugated to an isotype control Ab [35]. Generation of vaccines via this method is diagramed in Figure 1a. Similar technologies have been used to fuse influenza hemagglutinin (HA) to α-MHC-II Ab, α-CCR1/3/5 Ab [42], or cytokines [43,44]. In the case of cytokines, when HA was conjugated to Xcl1 (a ligand for Xcr1) or Ccl3 (a ligand for CCR1/3/5) the vaccines targeted different subsets of DCs and therefore resulted in increased CD4+ and CD8+ T cell proliferation or only increased CD4+ T cell proliferation, respectively [43]. This phenomenon further emphasizes the ability to tailor targeting of Ags to induce specific immune responses through conjugation to different peptides. Further, these enhanced immune responses discussed above were seen in the absence of conventional adjuvant; when tested, targeted immunogens without adjuvant, were similarly or more efficient than untargeted-Ag administered with traditional adjuvant.

Conjugate vaccines have also been targeted to complement receptors on various immune cells. Pioneering work by Dempsey et al. involved conjugating hen egg lysozyme (HEL) to complement protein C3d, a ligand for complement receptors 2 (CR2) and 3 (CR3). Mice immunized with pure (C3d)3-HEL produced higher α-HEL Ab titers than those immunized with HEL plus Freud’s complete adjuvant [84]. Targeting Ag to CRs is a concept that our lab has also explored and applied to whole cell vaccines which will be discussed later in this review. CR2 is primarily expressed on B cells and follicular dendritic cells (FDCs); its engagement facilitates Ag uptake and presentation by FDCs, and activation of B cells. In addition, C3d engagement of CR3 on APCs induces translocation to the lymph node and increased Ag presentation. C3d conjugation has been applied to bacterial [49] as well as viral peptides [50,82] via genetic engineering of plasmids encoding the fusion constructs. This concept has also been extended to the minimum CR2-binding region of C3d, p28, which also has shown promise as a method for increasing Ag-specific immune responses [83,85,86,87,88].

### 3.2. Glyconjugate Vaccines

In addition to proteins, Ags of interest for vaccination may also be lipoproteins, peptidoglycan, polysaccharides (PS), and other capsular material. Enhancement of the immune response to PS components has been achieved by linking them to carrier or stimulatory peptides [89]. PS are primarily recognized by Toll-Like Receptors 2 (TLR2) and 4 (TLR4) which are expressed on many immune cells [90,91]. Linking PS to stimulatory or targeted peptides increases their recognition by the immune system; especially through interaction with helper T cells [45,92,93,94]. This technique has been applied to various vaccines through chemical conjugation (Figure 1d), including the approved vaccines against *Neisseria meningitidis* (*Nm*) and *Streptococcus pneumoniae* (*Sp*) [45].

To simplify and expand chemical conjugation, PS have been conjugated to carrier proteins or lipids through the use of glycosylation processes within bacteria [46,95,96,97]. In this case, engineered strains of *Escherichia coli* (*Ec*) that express oligosaccharyltransferases (OTase) are utilized for vaccine production. OTase transfers PS to carrier proteins and these conjugates can then be harvested and purified from *Ec* (Figure 1b). An example of this conjugation technique involves Ags of *Klebsiella pneumoniae* (*Kp*), a multi-drug-resistant bacteria that produces substantial amounts of PS capsule which shields it from the immune system [98]. Development of a PS capsule-targeting immune response, as opposed to a protein-targeted response would be extremely advantageous for this pathogen. A bivalent vaccine against two *Kp* serotypes has been engineered by Feldman et al. using both K1 and K2 capsular PS locus clusters [46]. These PS were each produced by *Ec* which transferred them to exotoxin protein A from *Pseudomonas aeruginosa (Pa)* as a carrier. These purified K1 and K2 conjugates were then mixed and used to vaccinate mice; the bivalent vaccine induced increased α-*Kp* Ab levels as well as protection from lethal *Kp* infection.

### 3.3. Conjugated Nanoparticle-Based Vaccines

Nanoparticles are larger than subunit vaccines and have been shown to promote trafficking of Ag to lymph nodes [99]. NPs can be formed using a variety of materials including: self-assembling lipids (liposomes), sucrose polymers, gold, and proteins [100]. Peptide NPs may be loaded by using genetic techniques, leaving their self-assembling domains intact [101]. In addition, NPs may be decorated via chemical conjugation (Figure 1c). This process involves activation of the NP to reveal active residues that are then available for bond formation with moieties of interest [102]. For example, N-hydroxy-succinimide is used to activate primary amines in lysine residues [102,103]. This strategy was utilized to display *Plasmodium falciparum* protein Pfs25 on NPs to be tested as a malaria vaccine [47,104]. It was found that chemical conjugation of the Ag to the NP resulted in generation of more Abs, compared to genetic or SpyTag conjugation where “tag” and “catcher” peptides are added to the desired conjugates. In some cases, the conjugated cargo may be released from the NPs in a regulated manner. Schudel et al. recently utilized NPs to concentrate delivery at the lymph nodes where cargo was then released in phases due to the use of linkers with different half-lives [105]. Delaying the release of some cargo from NPs may allow for engagement of different lymphatic cell populations and has the potential to enhance immune responses and/or drug effects. In addition to targeting the lymph nodes, loaded NPs have also been further decorated with mannose [48] and anti-DEC-205 mAbs [106]; targeting them to mannose receptor on APCs or DEC-205 on DCs, respectively.

### 3.4. Targeting and Conjugation Involving Whole-Cell Vaccines

Pompa-Mera et al. created a plasmid which encoded a fusion protein of p28, a *Trichinella spiralis* (*Ts*) peptide, and a *Salmonella* outer membrane protein (OMP) with a proteolytic site between the OMP and p28-*Ts* peptide fusion [52]. The plasmid was then transformed into *Salmonella*, resulting in a strain that produced the OM-located fusion protein, from which the targeted *Ts* peptide was liberated. This strain was administered as a live vaccine to mice where it induced increased *Ts*-specific Ab levels and conferred protection against subsequent *Ts* challenge. This method of vaccine generation is diagramed in Figure 1e and combines the targeting ability of p28 with the generation of danger signals from a live infection.

Our lab similarly produced a CR-targeted vaccine via generation of a plasmid encoding a CR-targeting fusion protein that was transformed into bacteria which were then administered as a whole cell vaccine. In this case, we engineered a plasmid encoding C3d conjugated to YadA, a well-characterized *Yersinia* OMP without a cleavage site between the proteins. Therefore, transformation of bacteria with the plasmid resulted in expression of OM-localized, surface-displayed C3d. This promotes targeting of the entire bacterial immunogen to CRs on immune cells and enhances protective responses; we have demonstrated this utilizing murine pulmonary *Francisella tularensis* infection as a model [51]. This technique is diagramed in Figure 1f.

## 4. Summary

The ability to combine therapies or prophylactics with another element that facilitates delivery to their target has advanced a wide range of fields; including the treatment of cancers, dementia, and bacterial infections, as well as enhancement of vaccinations. Targeting has been achieved through bioconjugation of two or more moieties that embody different functions. Generally, one subunit will provide the targeting or enhancement function, whereas the other performs the desired treatment. This not only enhances treatment efficacy but also limits unwanted side effects; appealing traits to virtually all patient care. It is important to note however, that the potential for side effects may not be absolutely eliminated, even with targeting to distinct sites or cells. Another potential drawback in developing targeted treatments is the delay in approval for new treatments. In the case of cancer treatments, a broadly administered chemotherapy agent may be more readily available for patient care. Further, targeting therapies and prophylactic treatments to specific cell types may impede involvement of other cells whose benefits are not yet fully realized. These facts underscore the need for continued research and advancement of targeted therapies.

## Figures and Tables

**Table 1:** Examples of targeted therapies and vaccines.

Targeting Unit	Cargo	Targeted Disease/Treatment/Effect	Stage of Use
Cancer Therapies			
α-LIV-1 Ab	Monomethyl auristatin E	Metastatic breast cancer [15,16,17,34]	Phase 2 trials
α-HER2 Ab	Deruxtecan	HER2-positive breast & stomach cancer [15,18]	Clinical use
α-HER2 Ab	Mertansine	HER2-positive early breast cancer [23]	Clinical use
α-CD33 Ab	N-acetyl γ calicheamicin	Acute myeloid leukemia [24,25,35]	Clinical use
α-CD33 Ab	Calicheamicin	Relapsed/refractory acute myeloid leukaemia [24]	Clinical use
α-CD30 Ab	Monomethyl auristatin E	Hodgkin’s lymphoma [22]	Clinical use
α-CD22 Ab	Calicheamicin	Relapsed/refractory acute lymphoblastic leukaemia [25]	Clinical use
α-gp72 Ab	Monomethyl auristatin E	Models of ovarian and colon carcinoma [30]	Pre-clinical
**Central Nervous System Therapies**			
B6 peptide	Curcumin	Alzheimer’s disease. Delivery of loaded NPs to TfRs on the BBB, improved memory & learning [36]	Pre-clinical
TP10 peptide	Dopamine	Parkinson’s disease. Delivery of dopamine to the brain [37]	Pre-clinical
LJM3064 aptamer	Exosomes	Multiple sclerosis (EAE) associated demyelination [38]	Pre-clinical
α-PSA-NECM Ab	SC-79	Post-stroke neuro-regeneration. Delivery of SC-79 loaded NPs to neuroblasts to enhance pro-survival signaling [39]	Pre-clinical
**Antimicrobials**			
α-teichoic acid Ab	Rifalogue	Enhanced killing of intracellular MRSA [40]	Pre-clinical
α toxin-reactive NPs	Vancomycin	Controlled release of antibiotic at the site of infection [41]	Pre-clinical
**Targeted Vaccinations**			
α-Clec9A Ab	*Influenza* M2e	Target *influenza* Ag to DCs to enhance responses & protection [35]	Pre-clinical
α-MHC-II Ab	*Influenza* HA	Increased α-HA Ab & Th2 responses, protecting against *influenza* [42]	Pre-clinical
α-CCR1/3/5 Ab	*Influenza* HA	Increased CD8+ & Th1 responses, protecting against *influenza* [42]	Pre-clinical
Xcl1	*Influenza* HA	Increased proliferation of CD4+ & CD8+ T cells against *influenza* [43,44]	Pre-clinical
Ccl3	*Influenza* HA	Target HA to CCR1/3/5 to induce CD4+ T cells against *influenza* [43]	Pre-clinical
Diptheria toxoid	*Nm* PS	Increased α-*Nm* PS Abs, protection for meningococcal disease [45]	Clinical use
*Pa* exotoxin protein A	*Kp* PS	Increased α-*Kp* PS Abs, protection against *Kp* infection [46]	Pre-clinical
Qβ VLPs	Pfs25	Increased transmission-blocking Abs against malaria [47]	Pre-clinical
Mannose	*hepB* DNA	APC transfection via MR, stimulating α-HepB responses [48]	Pre-clinical
C3d	*Se* FimA	Increased immunogenicity of FimA, protection against *Se* [49]	Pre-clinical
C3d	HIV1 Env	Increased neutralizing Ab production against HIV1 [50]	Pre-clinical
C3d	*Ft* whole cells	Increased Ag binding to APCs, protection against tularemia [51]	Pre-clinical
C3d p28	*Ts* Ag30	Increased Ab production, protection against trichinosis [52]	Pre-clinical
